# Systemic redistribution of the intramyocardially injected mesenchymal stem cells by repeated remote ischaemic post‐conditioning

**DOI:** 10.1111/jcmm.13331

**Published:** 2017-09-25

**Authors:** Qin Jiang, Tao Yu, Keli Huang, Hao Zhang, Zhe Zheng, Shengshou Hu

**Affiliations:** ^1^ Department of Cardiac Surgery Sichuan Provincial People's Hospital Affiliated Hospital of University of Electronic Science and Technology Chengdu China; ^2^ State Key Laboratory of Cardiovascular Disease National Center for Cardiovascular Disease Fuwai Hospital Chinese Academy of Medical Sciences and Peking Union Medical College Beijing China

**Keywords:** Ischaemic post‐conditioning, Mesenchymal stem cell transplantation, Stromal cell derived factor‐1 alpha, Myocardial Ischemia, Cell distribution

## Abstract

We investigated the effect of repeated remote ischaemic post‐conditioning (RIPoC) on the organ distribution of the intramyocardially injected MSCs in rat myocardial ischemia model. Myocardial ischemia of adult female Sprague‐Dawley rats was induced by 30‐min. obstruction of the left anterior descending coronary artery. Repeated RIPoC was induced after ischemia with three cycles of 5‐min. occlusion and reperfusion of the limb with the frequency of half a day, 1 or 2 days, respectively. Compared with that by single RIPoC, repeated RIPoC transiently reduced oxidative stress, lipid peroxidation and inflammation in ischaemic myocardium; the gene expression of stromal cell‐derived factor‐1 alpha (SDF‐1α) was consistently induced by repeated RIPoC procedures. A total of 4 × 10^6^ male bone marrow‐derived MSCs were intramyocardially injected into ischaemic myocardium at 1 week after reperfusion. Three weeks later, immunohistological examination and quantitative reverse transcriptase polymerase chain reaction demonstrated that repeated RIPoC significantly increased MSCs retention in myocardium and decreased MSCs distribution over the lungs, spleen and liver; echocardiography assessment revealed that further cardiac function enhancement imposed by repeated RIPoC procedure. Furthermore, blockade with the anti‐CXCR4 antibody before cell transplantation markedly attenuated the benefits of therapeutic efficacy and cardiac function. Repeated RIPoC enhanced MSCs engraftment in ischaemic myocardium and reduced the distribution of MSCs over peripheral organs in a frequency‐effect way, but it reached a ceiling of maximal effect when applied with every 1 day. The SDF1α‐CXCR4 interaction played a major role in MSCs systemic redistribution induced by repeated RIPoC.

## Introduction

Mesenchymal stem cells (MSCs) transplantation delivered by intramyocardial injection showed promising benefits on left ventricular function recovery after myocardial ischemia 1. But its low engraftment in the heart was affected by harsh milieu resulting from ischemia reperfusion (IR) 2 and cell emigration from the injection site 3. After intravenous influx, these cells were widely distributed throughout the body, which were mostly entrapped in the lungs, spleen and liver. Moreover, cells migration from intramyocardial injection site neither resulted in deterioration of lungs, liver or spleen function, nor differentiated into fibroblast, myofibroblast or alveolar epithelial cells 4. Multimodal imaging showed MSCs transplantation increased myocardial viability and sustained engraftment without *de novo* cardiac differentiation 5. Thus, to recruit the entrapped or circulating MSCs into ischaemic myocardium was a feasible effort to improve therapeutic efficacy for myocardial infarction.

Ischaemic conditioning, this endogenous phenomenon could paradoxically protect the heart from acute myocardial infarction by subjecting it to one or more brief, non‐lethal episodes of ischemia and reperfusion 6. In the previous study, recruitment and mobilization of bone marrow‐derived stem cells had been proposed as conceivable mechanisms of ischaemic preconditioning to protect against IR injury at its late stage 7. We also concluded that RIPoC could induce transient gene expression of SDF‐1α and recruit intravenously infused MSCs into ischaemic heart 8. Additionally, RIPoC could enhance intramyocardial MSCs engraftment and therapeutic efficacy by ameliorating the deteriorated milieu in a rat model of acute myocardial ischemia 9.

It had been demonstrated that repeated RIPoC was not associated with a further reduction of infarct size for ischaemic preconditioning, but protected against adverse left ventricular remodelling and improved survival in a rat model of myocardial infarction 10. The objective of this study was to explore whether repeated RIPoC could enhance therapeutic efficacy of cell transplantation by protecting the survival in ischaemic myocardium for reduction of milieu damage, or recruiting the extracardiac escaped MSCs to the ischaemic heart and changing the MSCs distribution over systemic organs for steady induction of SDF‐1α expression. Meanwhile, it was also to explore whether the change of MSCs redistribution induced by repeated RIPoC procedure was attributable to SDF‐1α/CXCR4 interaction.

## Materials and methods

All the experiments conformed to the guidance for care and use of laboratory animals in accordance with the European Commission Directive 86/609/EEC (European Convention for the Protection of Vertebrate Animals used for Experimental and Other Scientific Purposes) and the UK Home Office (Scientific Procedures) Act (1986) with project approval from the Medical Ethics Committee of Sichuan Provincial People's Hospital & Affiliated Hospital of University of Electronic Science and Technology. The reference number for approving this study was 2016‐48.

### Animal preparation

Female SD rats (250–300 g) were used and anaesthetized by intraperitoneal injection of 10% chlora hydrate (3.5 ml/kg). Different concentration of isoflurane was inhaled through an oxygen mask depending on the surgical stage (2% during painful stimuli, and 1% during latent periods). The animals were orally intubated with a 23‐gauge vinyl catheter and ventilated *via* Harvard Model 683 Small Animal Ventilator (Harvard Biosicence Company, Holliston, MA, USA) with a tidal volume of 10 ml/kg and a ventilator frequency of 70 strokes per min.

### MSCs preparation

All the MSCs for engraftment experiments were harvested and cultured from 60 g male Sprague‐Dawley (SD) rats. In brief, the animals were executed by dislocating the neck. Bone marrow cavities of femur and tibia were flushed with culture media under sterile conditions. The adhesive MSCs were cultured to the third‐generation while the non‐adhesive cells were removed by changing fresh media. The expression of surface antigens was detected with CD29, CD45 and CD90 by flow cytometry (Abcam, Cambridge, UK). The images of MSCs morphology were detected with CD34, CD44 by immunofluorescence and white right. To trace the injected MSCs, the thymidine analogue 5‐bromo‐29 deoxyuridine (BrdU; Sigma‐Aldrich, St. Louis, MO, USA) was dissolved and added to culture medium overnight at a final concentration of 10 μmol/l.The amounts of MSCs were numerated with cell counting instrument (Countess; Invitrogen, Carlsbad, CA), and the viability of the injected MSCs was tested with typan blue (Invitrogen).

### Experimental design

**Figure 1:**
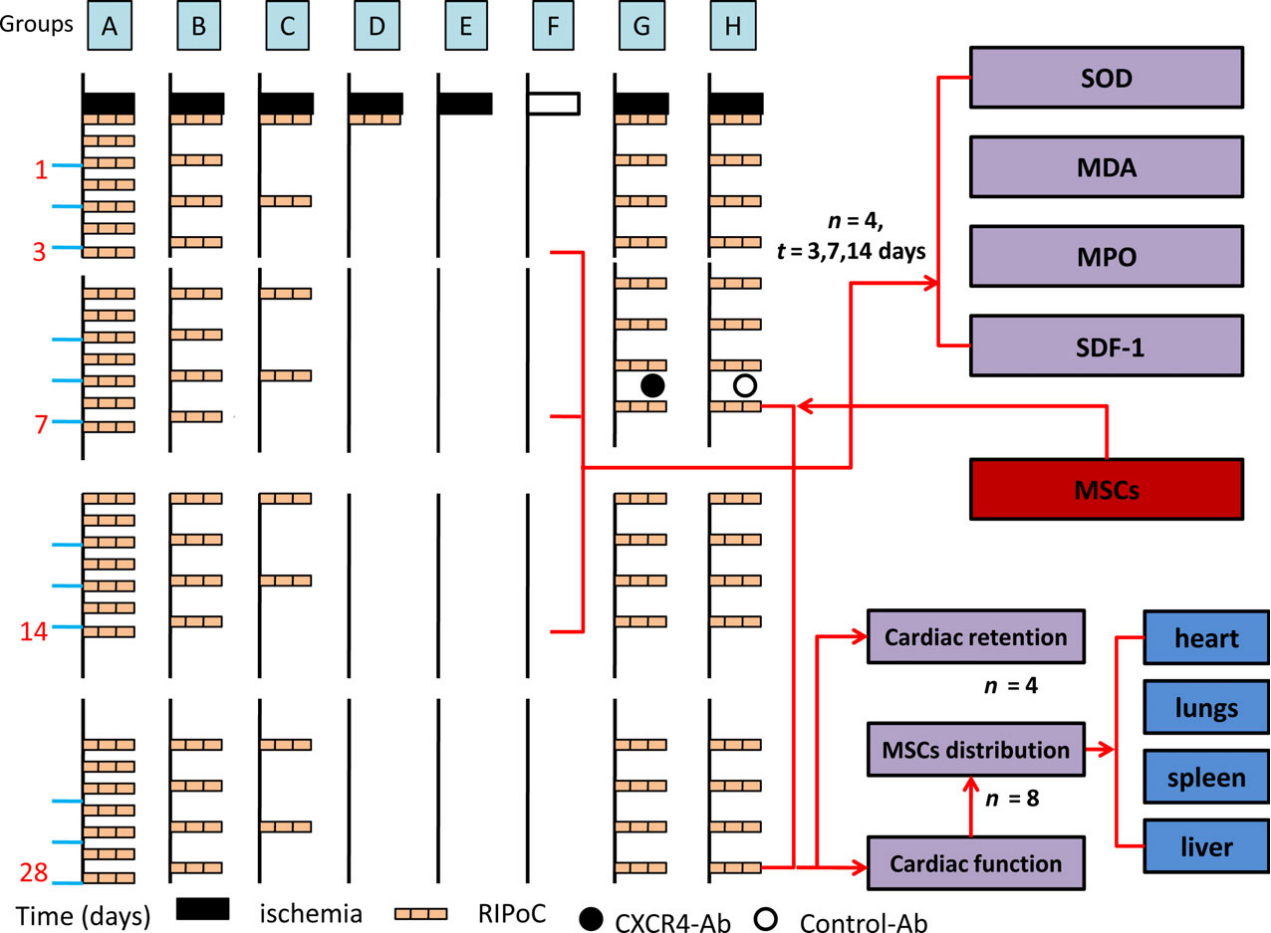
Study design. All groups but control group suffered myocardial ischemia induced by 30‐min. temporary obstruction on the left anterior descending artery (LAD). Repeated remote ischaemic post‐conditioning (rRIPoC) was induced by three cycles of 5‐min. occlusion and reperfusion of the limb with the frequency of every half day, every 1 day or every 2 days (A, B, C), respectively (*n* = 12). Remote ischaemic post‐conditioning (RIPoC) was induced by three cycles of 5‐min. occlusion and reperfusion of the limb (D). Ischemia reperfusion (IR) was induced in anaesthetized rats by LAD occlusion followed by uninterrupted reperfusion (E). The anaesthetized rats were only subject to open chest procedure as control group (Control, F). The effect on myocardium micro‐environment of rRIPoC procedure was detected from the sides of anti‐oxidative stress, lipid peroxidation, tissue inflammation cell infiltration and stromal cell‐derived factor‐1 alpha (SDF‐1α) gene expression at 3, 7, 14 days after reperfusion (*n* = 4). Injection of 4 × 10^6^ mesenchymal stem cells (MSCs) was intramyocardially administered at 1 week after the onset of reperfusion (*n* = 12). The effect on MSCs transplantation exerted by rRIPoC procedure was examined from cardiac contractile function and MSCs distribution over heart, lungs, spleen and liver at 21 days after cell transplantation. Another two groups the rats were intraperitoneally administered the CXCR4 specific antibody (G) or control antibody(H) before cell transplantation in the group, which resulted in the maximum SDF‐1α effect (*n* = 12). SOD: superoxide dismutase; MDA: malondialdahyde; MPO: Myeloperoxidase.

Animals subject to ischemia received different modes of reperfusion as illustrated in Figure 1. Left thoracotomy was performed, and myocardial ischemia/reperfusion was induced by 30‐min. temporary obstruction of left coronary artery as described before 8. In brief, left anterior descending artery (LAD) 2 mm distant from tip of the normally positioned left auricle was tied with reversible knot by a 6‐0 polyester suture attached to a small curved needle. RIPoC group received three cycles of 5‐min. ischemia and reperfusion on the limb with tourniquet at the onset of reperfusion. Repeated RIPoC (rRIPoC) groups received the same RIPoC procedure on the same limb with three distinct frequency cycles, ranging from with every half day, every 1 day, to every 2 days, respectively. IR group (no intervention in the whole reperfusion period). Control group (only open chest surgery). Intramyocardial injection of the MSCs around the ischaemic borderline was administered at 1 week after reperfusion. The CXCR4‐Ab group received an intraperitoneal injection of 10 μg/kg rabbit anti‐rat CXCR4 monoclonal antibody (Abcam) with the maximum SDF‐1α effect before MSCs transplantation to testify the role of SDF‐1/CXCR4 axis, and administration of the same dose of a non‐specific antibody was designated as control‐Ab group 7. The hearts from each groups were harvested at 3, 7, 14 days after myocardial ischemia (*n* = 4) or 21 days after MSCs transplantation (*n* = 12).

### The indexes of IR injury

To assess the effect of distinct ischaemic post‐conditioning strategies after myocardium ischemia, the indexes of anti‐oxidative stress, lipid peroxidation and tissue inflammation cell infiltration were compared at 3, 7 and 14 days among the six groups with their presumptive indicators, respectively (*n* = 4) 9. The borderline zone of ischaemic myocardium (2 mm width equally spanned on normal‐dilated myocardium borderline) was harvested and homogenized. The enzymatic activities of superoxide dismutase (SOD, expressed as U/mg protein), malondialdahyde (MDA, expressed as nmol/mg protein) and Myeloperoxidase (MPO, expressed as μUnit/mg protein) were measured spectrophotometrically at the recommend wave length according to the manufacturer's instructions (Nanjing Jiancheng Bioengineering Institute, Nanjing, China) as previously described.

### SDF‐1α gene expression levels in myocardium

To examine the gene transcription expression of SDF‐1α, the borderline zone of ischaemic myocardium was harvested at 3, 7 and 14 days after the procedure (*n* = 4) 8. Total RNA was isolated using TRIzol Reagent (Invitrogen). For reverse transcription polymerase chain reaction (PCR), 1 μg of total RNA was added to the reverse transcription system (Promega, Madison, WI, USA). The reaction conditions were denaturation for 10 min. at 70°C followed by 15 min. at 42°C and 5 min. at 95°C. The resulting cDNA was used as the template in PCR with cDNA‐specific primers spanning at least one intron. The following oligonucleotides were used as primers (Takara Biotechnology Co., Dalian, China): for SDF‐1α: Forward, 5′‐TGAGAGCCATGTCGCCAGA‐3′, and Reverse, 5′‐GGATCCACTTTAATTTCGGGTCAA‐3′; for GAPDH: Forward, 5′‐GGCACAGTCAAGGCTGAGAATG‐3′, and Reverse 5′‐ATGGTGGTGAAGACGCC AGTA‐3′. For quantitative real‐time PCR, 4 μl cDNA diluted 20‐fold was used with Master Mix SYBR Green One (Applied Biosystems, Carlsbad, CA, USA) and normalized to GAPDH.

### Immnohistochemical analysis of BrdU‐labelled MSCs retention in heart

Slides for immnohistochemical examination were prepared from formalin‐fixed and paraffin‐embedded hearts. Mouse monoclonal anti‐BrdU antibody (Sigma‐Aldrich) was used to detect the labelled cells as previously described (*n* = 4) 9. The cells with brown‐stained nuclei were directly counted under a microscope (Olympus BX61, Tokyo, Japan) at 400‐fold magnification in ten positive fields from three distinct slides of each heart.

### Real‐time PCR analysis of MSC retention

For absolute quantification of engrafted MSCs, the organs were harvested at 3 weeks later after cell transplantation as previously described (*n* = 8) 4, 8, 9. The genomic DNA was isolated from the whole heart, lungs, spleen and liver. Standard curve was generated by five samples of 10‐fold serial dilution of 10^5^ male cultured MSCs. A certain percentage of tissue and abstracted DNA was used into the reaction system for ultimately calculating the total number of implanted MSCs on the specific organ. The primer of SRY gene for detecting Y chromosome was as followed: Forward, 5′‐ CATCGAAGGGTTAAAGTGCCA‐3′; and Reverse, 5′‐ATAGTGTGTAGGTTGTTGTCC‐3′. The real‐time PCR conditions were an initial denaturation step of 10 min. at 95°C, followed by 40 cycles of 95°C for 15 sec. and 60°C for 1 min. on the ABI PRISM 7300 sequence detection system (Applied Biosystems). A dissociation curve was generated for all reactions, and one single crest verified the presence of primer specific amplification.

### MSCs distribution in peripheral organs

For absolute quantification of engrafted MSCs in peripheral organs on which MSCs were prone to reside, the lungs, spleen and liver were also harvested at 3 weeks later when the heart was picked out (*n* = 8) 4. The number of MSCs in their respective organs was detected by real‐time PCR as performed on heart, respectively. The percentage of the MSCs amount in specific organ to the total injected number was recorded.

### Cardiac function

A specialized sonographer who was blinded to the experimental design utilized echocardiography (UCG) to record cardiac function 3 weeks after cell transplantation (*n* = 12). The left ventricular end‐diastolic diameters (LVEDD) and left ventricular end‐systolic diameters (LVESD) were measured from M‐mode recordings. Left ventricular fractional shortening (LVFS) was calculated as: (LVEDD‐LVESD) × 100/LVEDD 4, 8, 9. The results were the average of six selected cardiac cycles from two independent scans. Dimensions were measured between the anterior wall and the posterior wall in the short‐axis view with papillary muscles as the reference point.

### Data and statistical analysis

All data were presented as mean ± S.D. Statistical analyses were performed using SPSS 17.0 (SPSS, Inc., Chicago, IL). Statistical significance was evaluated with one‐way anova for multigroup comparisons, followed by LSD test to determine where the significance lay. Differences were considered as statistically significant when *P* values less than 0.05.

## Results

### MSCs preparation and experimental model mortality

**Figure 2:**
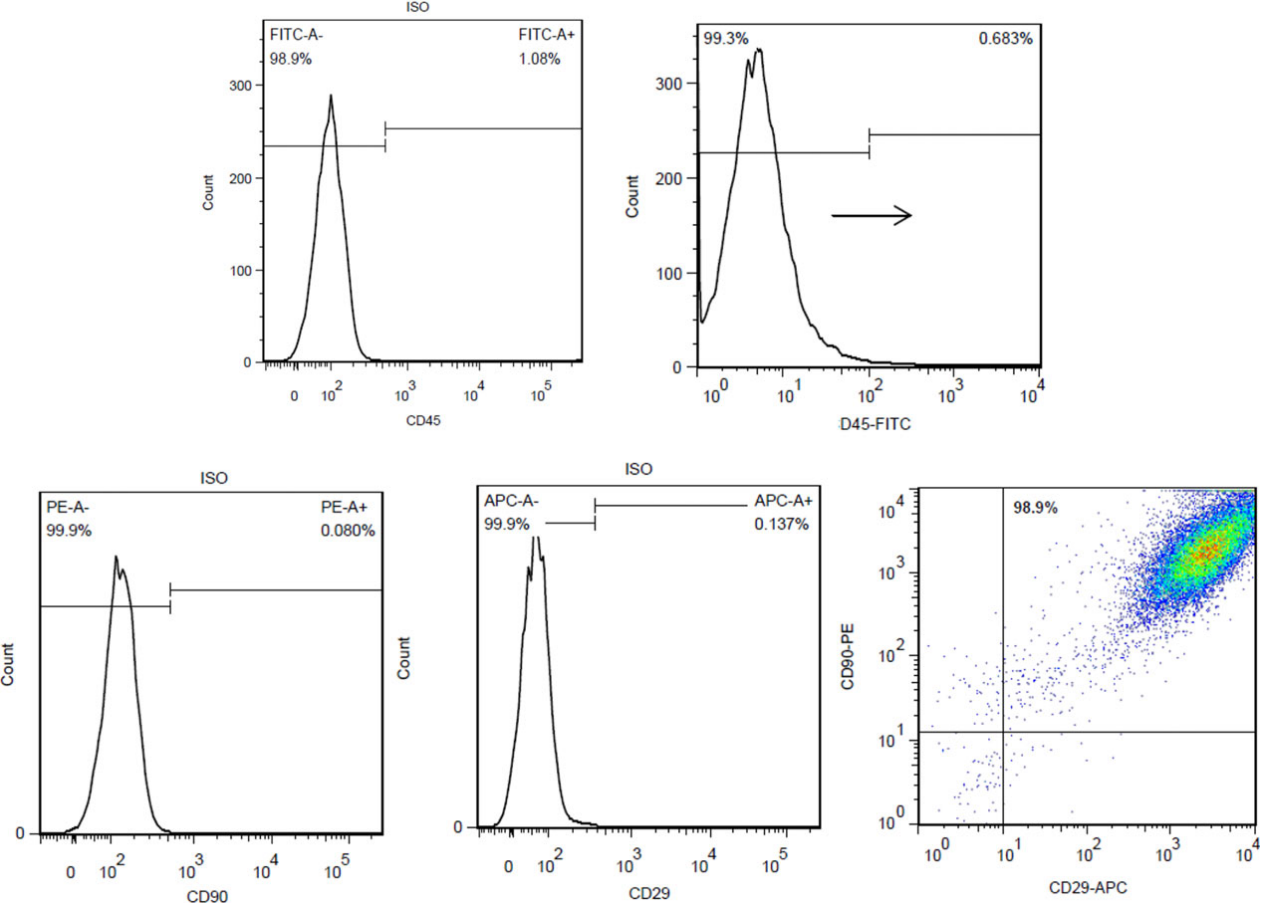
The identification of MSCs surface antigen expression. The Figure 2 shows the results with CD45^−^CD90^+^CD29^+^ by flow cytometry.

**Figure 3:**
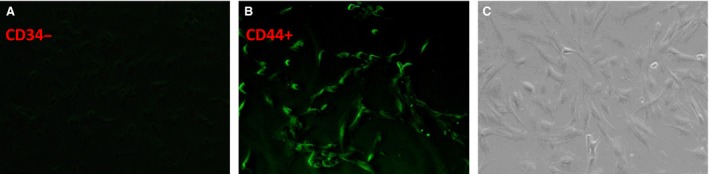
The detection of MSCs morphology. **A**,** B** shows the result of CD34^−^CD44^+^ by immunofluorescence, respectively. The **C** was the white light images on MSCs morphology.

The expression of surface antigens was CD45^−^CD90^+^CD29^+^ by flow cytometry (Fig. 2). MSCs showed the CD34^−^CD44^+^ by immunofluorescence (Fig. 3A and B). MSCs formed patchy colonies with spindle‐shaped fibroblast‐like appearance in culture plates (Fig. 3C). Both the efficiency of BrdU labelling and the viability of MSCs by trypan blue test for transplantation were nearly 100%. On the basis of the experimental protocol, one hundred and seventy‐eight rats were used in this study. The death of 10 rats mainly happened during cardiac ischemia and MSCs injection period. No death event occurred during completion of rRIPoC procedures.

### The SOD level for the anti‐oxidative stress injury

**Figure 4:**
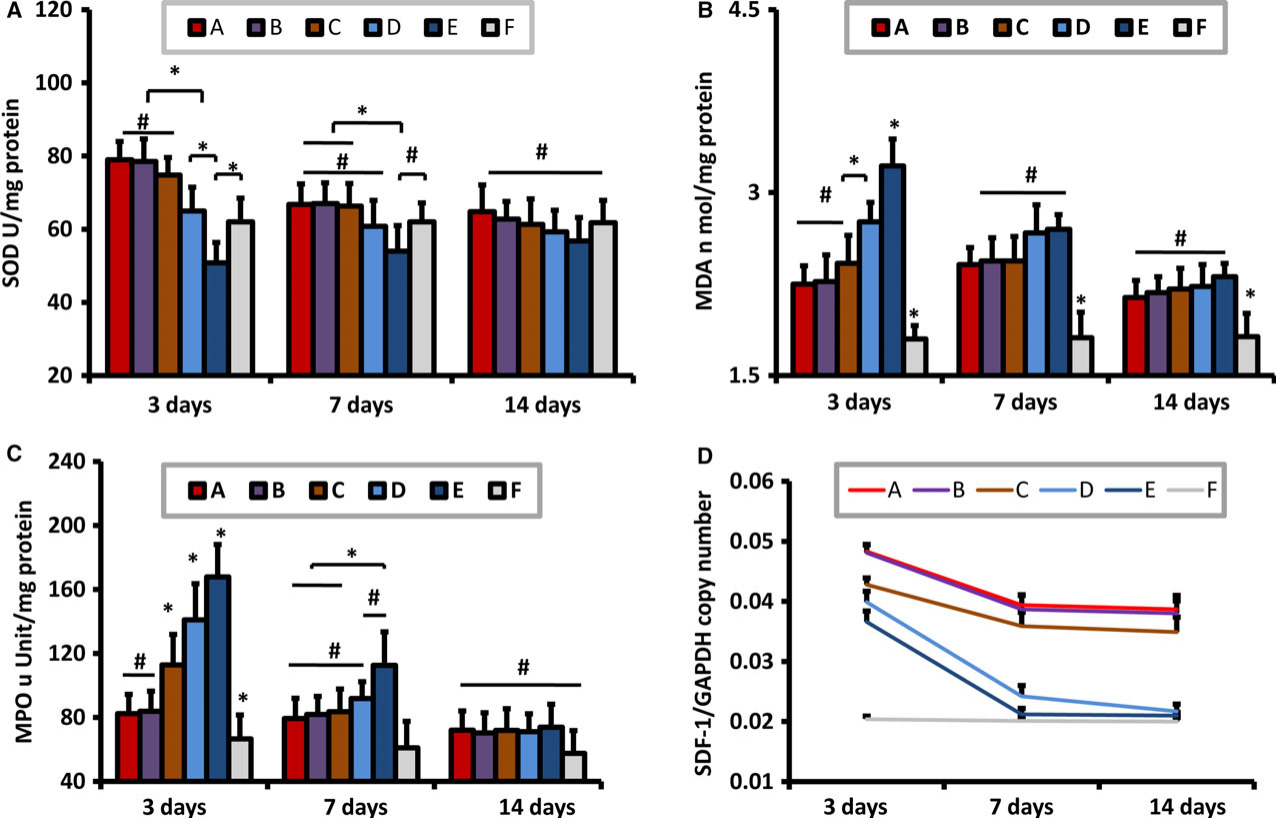
The level of SOD, MDA, MPO and SDF‐1α expression in myocardium with relationship of repeated remote ischaemic post‐conditioning (rRIPoC). Repeated remote ischaemic post‐conditioning (rRIPoC) with three different frequency reduced the SOD level (**A**), MDA content (**B**) and MPO level (**C**) compared with RIPoC procedure at 3 days after ischemia (*P *<* *0.05). But there were no significant changes in the three repeated RIPoC groups compared with these in RIPoC group at 7, 14 days after ischemia, respectively. The level of stromal cell‐derived factor‐1 alpha (SDF‐1α) gene expression was up‐regulated in ischaemic myocardium by repeated RIPoC procedure compared with that in RIPoC at 3, 7 14 days after ischemia, respectively (*P *<* *0.05, **D**). There was presumed dose–effect escalation relationship in SDF‐1α on the three repeated RIPoC groups. But it did not change when applied every half day compared with that for with every 1 day. *n* = 4. The abbreviations are the same as in Figure 1 legend. *P <* *0.05; #: not significant.

The level of SOD in IR group was most reduced at 3 days after ischemia compared with other groups (*P *<* *0.05), and recovered gradually at 7 and 14 days after reperfusion. RIPoC remained at transiently increased level, and enhanced the SOD with 65.0 ± 6.5 U/mg protein at 3 days after ischemia (*P *<* *0.05), but comparable with that in IR group at 7 and 14 days after ischemia. rRIPoC raised the SOD level at 3 days after ischemia compared with that in RIPoC procedure, with 79.0 ± 5.0 U/mg protein for every half day, 78.5 ± 6.2 U/mg protein for every 1 day, 74.8 ± 4.8 U/mg protein for every 2 days (*P *<* *0.05, *versus* RIPoC). The rRIPoC procedures underwent the decreased SOD level at 7 days after ischemia similar with RIPoC procedure, but had higher SOD level compared with that in IR group with 66.8 ± 5.6 U/mg protein for every half day, 67 ± 5.7 U/mg protein for every 1 day, 66.3 ± 6.2 U/mg protein for every 2 days (*P *<* *0.05, *versus* IR). There was no significant difference in SOD level at 14 days after reperfusion among all the groups. There was no statistical increase in SOD level when applied with every half day compared with that with every 1 day at all the three time‐points (Fig. 4A).

### The MDA level for the lipid peroxidation injury

The level of MDA content in IR group was most increased at 3 days after ischemia compared with that in other groups (*P *<* *0.05), and fell gradually at 7 and 14 days after reperfusion, but higher than that in control group (*P *<* *0.05). RIPoC only reduced the MDA content at 3 days after ischemia with 2.76 ± 0.16 nmol/mg protein (*P *<* *0.05), comparable with that in IR group at 7 and 14 days after ischemia. The three rRIPoC groups had the similarly decreased MDA content compared with that in RIPoC group at 3 days after ischemia with 2.25 ± 0.15 nmol/mg protein for every half day, 2.27 ± 0.22 nmol/mg protein for every 1 day, 2.42 ± 0.23 nmol/mg protein for every 2 days, but comparable with that in RIPoC group at 7 and 14 days after ischemia (Fig. 4B).

### The MPO level for tissue inflammation reaction

The level of MPO in IR group was most enhanced at 3 days after ischemia compared with that in all the other groups (*P *<* *0.05), fell down at 7 days after reperfusion (*P *<* *0.05), and recovered back to baseline at 14 days after reperfusion (*P *>* *0.05). RIPoC procedure reduced the MPO compared with that in IR group only at 3 days after ischemia (*P *<* *0.05). The three distinct rRIPoC procedures reduced the MPO level compared with that with RIPoC procedure at 3 days after ischemia, with 82.3 ± 12.1 μUnit/mg for every half day, 83.8 ± 12.6 μUnit/mg for every 1 day, 112.8 ± 19.1 μUnit/mg for every 2 days (*P *<* *0.05, *versus* 141.0 ± 22.6 μUnit/mg). The MPO level of the three rRIPoC groups was comparable with that in RIPoC group at 7, 14 days after ischemia, respectively (*P *>* *0.05; Fig. 4C).

### Sustained gene expression of SDF‐1α induced by rRIPoC

As the previous study showed, SDF‐1 gene expression relative to reference gene was increased after cardiac ischemia with 79.4% at 3 days after ischemia (*P *<* *0.05), normalized at 7 days after ischemia. RIPoC relatively enhanced SDF‐1 gene expression with 95.6% at 3 days after ischemia (*P *<* *0.01), with 20.4% at 7 days after ischemia (*P *<* *0.05), returned to normalization at 14 days. The three distinct rRIPoC methods induced a sustained rise of SDF‐1α mRNA expression in the myocardium, of 32.0% increase for every half day, 31.4% for every 1 day and 16.9% for every 2 days compared with that in IR group at 3 days after ischemia (*P *<* *0.01); 85.8%, 82.5%, 69.3% at 7 days after ischemia (*P *<* *0.01); 81.7%, 78.4%, 63.8% at 14 days after ischemia, respectively (*P *<* *0.01). There were no remarkable changes at various time‐points in the sham group (Fig. 4D).

### MSCs retention increase in IR myocardium by rRIPoC

**Figure 5:**
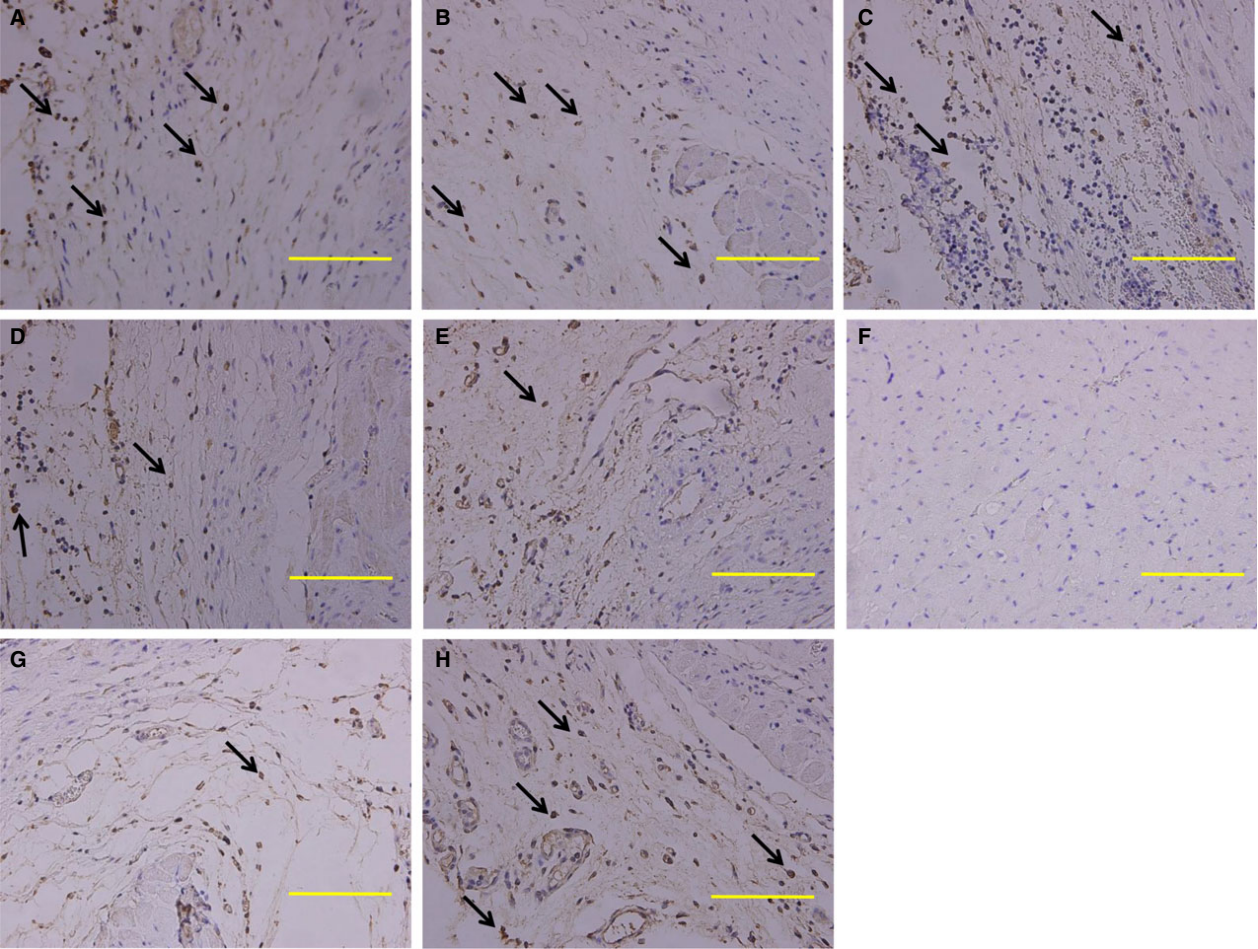
Immunohistochemical staining on MSCs retention in myocardium. Immunohistochemical staining examination showed the retention of BrdU‐labelled MSCs (arrow, the cells with brown‐stained nuclei) was significantly more in the three repeated RIPoC groups (**A**,** B**,** C**) than that in RIPoC group (**D**) or IR group (**E**) at 21 days after cell intramyocardial delivery. Scarcely BrdU‐labelled MSCs were found in sham group (**F**). It seemed that MSCs retention in the heart was in positive relationship with the frequency of repeated procedure. But MSCs retention in myocardium did not change when repeated RIPoC procedure applied every half day (**A**) compared with that with every 1 day (**B**). Administration with CXCR‐4 antibody (**G**) reduced the MSCs retention in myocardium at the maximum SDF‐1α effect of repeated RIPoC procedure (*P *<* *0.05). Administration of Control antibody (**H**) did not affect the MSCs retention in myocardium induced by repeated RIPoC procedure. The abbreviations are the same as in Figure 1 legend. Bar indicated 100 μm. The magnification was ×400 (*n* = 4).

**Figure 6:**
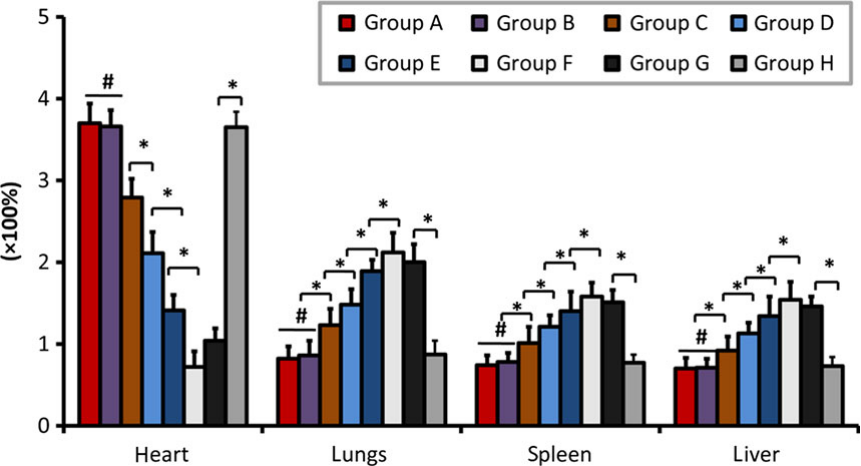
Repeated remote ischaemic post‐conditioning changed MSCs distribution over the heart and extracardiac organs. Polymerase chain reaction for the SRY gene, as a marker of the presence of the Y chromosome showed a significantly higher percentage of infused MSCs in repeated RIPoC group (A, B, C) compared that in RIPoC group (D) or IR group (E) at 21 days after intramyocardial delivery (*n* = 8). MSCs retention in the heart was inversely correlated with these in extracardiac organs, which was affected by repeated remote ischaemic post‐conditioning. Meanwhile, administration of CXCR‐4 antibody (G) but not control antibody (H) reduced the MSCs retention in myocardium but increased the distribution over extracardiac organs by repeated RIPoC procedure compared with that in group B with the presumed ceiling of the maximal SDF‐1α gene expression (*P *<* *0.05). The abbreviations are the same as in Figure 1.

There was a remarkable difference in the amount of brown‐staining cells (arrow) among the three rRIPoC groups, RIPoC group, IR group and control group (Fig. 5A–F). The number of Brdu‐labelled positive cells in RIPoC group (Fig. 5E) was significantly higher than that in IR group (Fig. 5D). The three rRIPoC groups (Fig. 5A–C) induced more MSCs retention in myocardium compared with that in RIPoC group with the seemed trend in a frequency‐effect escalation way, but no significant difference between group A and group B. For determining the accurate amount of engrafted MSCs in the seven groups, the real‐time PCR for the male‐specific SRY gene copy number was used to calculate the total number of the whole heart. There was higher percentage of engrafted MSCs in rRIPoC groups than that in RIPoC group (3.70 ± 0.24%, 3.66 ± 0.20% and 2.79 ± 0.23% *versus* 2.11 ± 0.26%, respectively, *P *<* *0.05; Fig. 6).

### MSCs retention in peripheral organs reduced by rRIPoC procedure

Contrary to MSCs retention in heart, the retention in lungs was negatively changed by myocardial ischemia or limb ischaemic conditioning, with 1.48 ± 0.19% in RIPoC group, 1.89 ± 0.14% in IR group, 2.12 ± 0.24% in control group, respectively (*P *<* *0.05). Compared with MSCs distribution in RIPoC group, there was lower MSCs retention in lungs of the three rRIPoC groups, with 0.82 ± 0.15% in group with every half day, 0.86 ± 0.18% in group with every 1 day, 1.23 ± 0.20% in group with every 2 days, respectively (*P *<* *0.05). But it seemed to be of frequency‐effect change among the three rRIPoC groups, but the minimum MSCs retention amount over peripheral organs attained on when rRIPoC was applied with every 1 day. Similarly, the MSCs retention in spleen and liver among the groups was also distributed like that in lungs. In all, it seemed that the MSCs distribution over peripheral organs was contrary to these over the heart, all the three rRIPoC groups had significant decrease of MSCs retention in extracardiac organs (Fig. 6).

### Role of SDF‐1α in rRIPoC‐induced MSCs organ redistribution

To testify the role of SDF‐1 in group G, we intraperitoneally administered the anti‐CXCR4 antibody on the rat, of which rRIPoC was administered with the frequency of every 1 day that presumed to be up to the maximal effect in transplantation efficacy (Fig. 4), which specifically blocks binding of SDF‐1 to its endogenous receptor CXCR4, diminished MSCs recruitment after rRIPoC with every 1 day by 71.6% (1.04 ± 0.15% *versus* 3.66 ± 0.20%, *P *<* *0.05). However, non‐CXCR4 specific antibody administration did not change the retention in IR heart induced by rRIPoC with every 1 day (3.65 ± 0.19%). Whereas that change was also accompanied with increased percentage in lungs, spleen and liver in rRIPoC + CXCR4 group (2.00 ± 0.22%, 1.51 ± 0.15%, 1.46 ± 0.12%) compared with these(0.86 ± 0.18%, 0.78 ± 0.11%, 0.71 ± 0.11%) in the rRIPoC group with the frequency of every 1 day, respectively (*P *<* *0.05, Figs 5G, 6).

### Heart function modified by rRIPoC procedure

**Figure 7:**
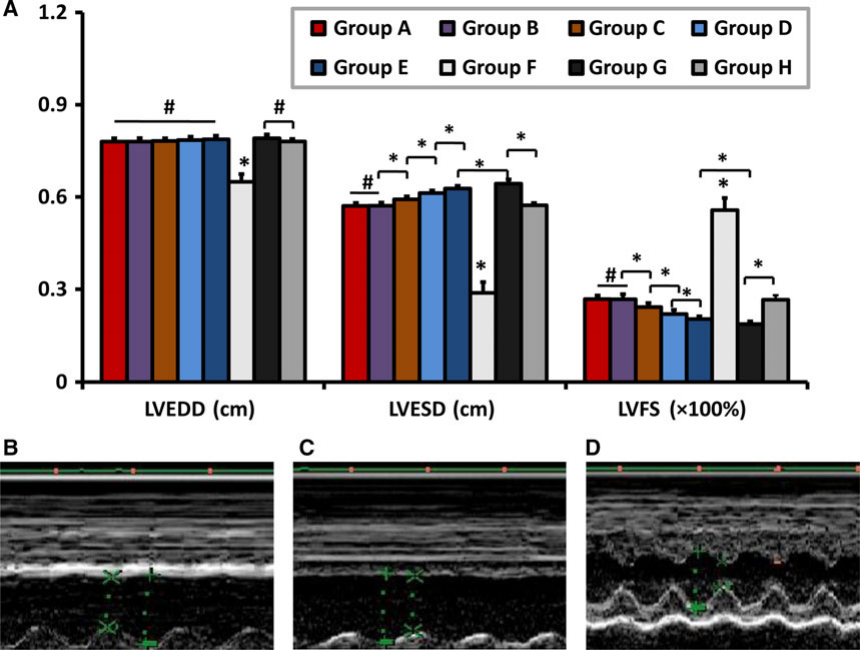
Echocardiographic measurements at 21 days after cell transplantation. The Figure 6A shows the indexes of cardiac function LVEDD, LVESD and LVFS after treatment. At 21 days after cell transplantation, the sham group (F) had only significantly lower value in LVEDD among all the groups (*P *<* *0.05). LVESD were significantly lower in the repeated procedure groups (A, B, C) than that in RIPoC group (D) and IR group (E), respectively. Repeated RIPoC procedure‐treated groups had significantly greater value in LVFS than that in RIPoC group (D) and IR group (E), respectively. The ameliorated value of LVESD and LVFS were associated with the number of repeated procedure compared with that in RIPoC group. But that values did not change when repeated RIPoC procedure applied every half day (**A**) compare with that with every 1 day (**B**). Meanwhile, administration of CXCR‐4 antibody (G) but not control antibody (H) decreased the cardiac function parameter by repeated RIPoC procedure (*P *<* *0.01), even than that in group E(*P *<* *0.05), which indicated that the contribution from MSCs retention was important than that from the repeated RIPoC procedure. The graphs **B**,** C** and **D** represented echocardiography images which reflected the diastolic and systolic function in group B, E and F, respectively. All values are mean ± S.E.M. (*n* = 12). *P <* *0.05. LVEDV, left ventricular end‐diastolic diameter; LVESV, left ventricular end‐systolic diameter; LVFS, left ventricular fractional shortening; other abbreviations are the same as in Figure 1 legend.

There were significant changes of heart function parameters in all of experimental groups compared with control group (*P *<* *0.05). LVESD was significantly reduced in the three rRIPoC groups in comparison with that in RIPoC group (5.71 ± 0.10 mm, 5.72 ± 0.10 mm and 5.93 ± 0.10 mm *versus* 6.13 ± 0.09 mm, *P *<* *0.05) at 21 days after MSC delivery. LVFS was higher in the three rRIPoC groups in comparison with that in RIPoC group (26.8 ± 1.2%, 26.7 ± 1.7%, and 24.2 ± 1.3%, *versus* 21.9 ± 1.4%, *P *<* *0.05). Blockade with CXCR4‐Ab worsened the cardiac function parameters ameliorated by rRIPoC procedure (LVESD: 6.43 ± 0.14 mm, LVFS: 18.7 ± 0.09%; *P *<* *0.01) and was also inferior to that in IR group (LVESD: 6.28 ± 0.09 mm, LVFS: 20.3 ± 0.09%; *P *<* *0.05), but the control‐antibody group did not affect the results (LVESD: 5.73 ± 0.10 mm, LVFS: 26.6 ± 1.4%; *P *>* *0.05). LVEDD showed no significant difference in any of the cell transplantation groups except that in sham group (Fig. 7A). The representative echocardiography images reflected the diastolic and systolic function in group B, group E and group F, respectively (Fig. 7B–D).

## Discussion

In this study, we showed that rRIPoC procedure enhanced the retention of the intramyocardially injected MSCs in rat IR model compared with single RIPoC. Enhanced efficacy on MSCs retention along with relative decrease on extracardiac organs induced by rRIPoC procedure was critically correlated with the frequency of ischemia conditioning after reperfusion. This effect on MSCs retention rendered by periodical rRIPoC administration which was blocked by CXCR4‐specific antibody, thus SDF‐1/CXCR4 homing signalling dominated to recruit the MSCs back to myocardium from systemic distribution after intramyocardial injection.

Cell‐based treatment raised great expectations on the evolution of biological therapy for regenerative medicine 11. Among the cell types under investigation, MSCs are most intensely studied for their unique properties, their availability and their key role in maintaining and replenishing endogenous stem cell niches, and in early stage, clinical studies show promise for repair of cardiac tissues 2. Accumulating data from preclinical and early phase clinical trials documented their safety when delivered as either autologous or allogeneic forms in a range of cardiovascular diseases 12. Comparative analysis in dose‐escalation clinical trial indicated that the chronic heart failure patients benefit from high dose of allogeneic mesenchymal precursor cells 13.

Although MSCs intramyocaridal transplantation is most efficacious compared with other delivery routes, the engraftment is quite limited 14. Apart from the necrotic/apoptotic signals in the inflammatory microenvironment where acute myocardial infarction happened, cell evasion from the injected site is also the dominant opposing force to the therapeutic activities of MSCs. The low survival of grafted cells may limit their therapeutic impact, whereas their distribution over other organs must be considered in all cell therapy applications 15. The percentage of donor cells in the heart decreased rapidly from 34% to 80% of injected cells (0 hr) to 0.3–3.5% (6 weeks) independent from cell type, number and application time 3. Continuous effort to further enhance the therapeutic benefit of MSCs is under way 16. Compared with medication‐conditioning approach, we were devoted to a non‐invasive and clinically available method to improve stem cell therapeutic efficacy.

In remote ischaemic conditioning (RIC), brief, reversible episodes of ischemia with reperfusion in one vascular bed, tissue or organ confer a global protective phenotype and render remote tissues and organs resistant to ischemia/reperfusion injury 17. The cardioprotection afforded by RIC is mediated *via* a complex mechanism involving sensory afferent nerves, the vagus nerve and release of a humoural blood‐borne factor 18. We showed in the previous study that RIPoC alleviated the ischemia‐reperfusion injury and ameliorated the retention of the intramyocardially injected MSCs in rat heart by modifying tissue microenvironment which suffered acute myocardial ischemia 9. This study also demonstrated the existence of a tissue microenvironment where oxidative stress, lipid peroxidation and inflammation reaction indexed by SOD, MDA, MPO, which was demonstrated that influenced cell survival, tissue repair and cardiac function recovery^9^, changed in a temporal and spatial manner. Repeated RIPoC procedure resulted in beneficial effect in terms of increasing anti‐redox reaction, reducing lipid peroxidation and tissue inflammation cell infiltration at 3 days after reperfusion. However, its effect seemed to be up to the ceiling when RIPoC was administered intermittently with every 1 day.

At the onset of reperfusion, opening of mitochondrial permeability transition pore (MPTP) in the inner mitochondrial membrane acts as a key nodal point in mediating cardiac dysfunction and cell death 19. A recent study in a rat IR model showed ischaemic post‐conditioning applied 60 min. after reperfusion was ineffective 20. Delayed coronary reperfusion seemed to be ineffective at impeding the dynamic increase in cardiac efferent sympathetic nerve activity following myocardial ischemia 21. Thus, the time window for protective intervention(s) during reperfusion has important implications in terms of clinical usefulness of ischaemic post‐conditioning. The cardioprotection against ischemia/reperfusion injury in rat by ischaemic post‐conditioning applied as late as 1 hr after the onset of reperfusion would be of no efficacy. In this experiment, we observed some differences in ischaemic myocardium only at 3 days after reperfusion between RIPoC and repeated RIPoC on the indexes of anti‐redox reaction, lipid peroxidation and inflammation cell infiltration reaction. Therefore, the transient effect could not afford the consistently assisted effect on the therapeutic efficacy of cell transplantation. We postulated that there was another signal pathway evoked by repeated RIPoC procedure which could causally affect the MSCs distribution.

Ischemia conditioning stimulates cardioprotection *via* an unidentified humoural factor, believed to be a protein between 3.5 and 15 kD. Stromal cell‐derived factor‐1 (SDF‐1α) is a chemokine of 10 kD that is induced by hypoxia and recruits stem cells, but also exerts direct, acute cardioprotection *via* its receptor, CXCR4 22. However, the molecule has short half‐live *in vivo* for the serum dipeptidase DPPIV cleaves and inactivates SDF‐1α. A hydrogel delivery system that sustains the release of the engineered stromal cell‐derived factor analogue (ESA) was developed to induce continuous homing of endothelial progenitor cells and improved left ventricular function in the model of myocardial infarction 23, 24. In the acute myocardial infarction, the presence of homing signals characterized by SDF‐1α/CXCR4 axis may be of benefit for cardiac repair 25. In the previous study, we also concluded that RIPoC could induce transient gene expression of SDF‐1α on the chronic infarction period and establish an SDF‐1α‐dependent gradient for stem cell recruitment and homing 8. However, the SDF‐1α expression after RIPoC was also seen in a time course like that change after MI 26, which was both transiently up‐regulated and thereafter down‐backed to baseline. Therefore, SDF‐1α could be theoretically kept at a higher level if RIPoC was serially repeated with specific frequency. In fact, SDF‐1α gene expression could be consistently enhanced before it downed back to baseline by repeated RIPoC procedure with the frequency of every 2 days in this experiment. Moreover, its gene expression level could be relatively raised with an intensified frequency up to every 1 day. But it seemed that the effect arise a ceiling when the frequency was up to every 1 day.

Stromal cell‐derived factor‐1 alpha was demonstrated as a chemokine that delivered a two‐pronged defence of the myocardium 27. The production of SDF‐1α in infarcted myocardium in the chronic phase of MI was associated with LV adverse remodelling and progressive dysfunction in AMI survivors 28. Whereas repeated remote post‐conditioning further reduces adverse LV remodelling and improves survival in a dose‐dependent fashion 10. Repeated RIC prevented the deterioration of LV diastolic function, reduced adverse LV interstitial fibrosis and oxidative stress by MI 29. Thus, the effect of left ventricular remodelling by ischaemic conditioning and its SDF‐1α product competed against each other. The ultimate effect remained to be determined in the future research.

In clinical trials, the cardiac systolic function recovery is usually the primary end‐point, also as the pivotal index of MSCs transplantation efficacy for myocardial ischemia. Our main purpose was to advance the amount of the intramyocardially injected MSCs in myocardium 3 weeks after transplantation. In the past decade, substantial evidence supports the paradigm that stem cells exert their reparative and regenerative effects, in large part, through the release of biologically active molecules acting in a paracrine fashion on resident cells 30. The percentage relative to the initial number of injected cells in heart, lungs, spleen and liver was 0.72 ± 0.19%, 2.12 ± 0.24%, 1.58 ± 0.17% and 1.54 ± 0.22% in control group, respectively. The results demonstrated that the detected MSCs were mainly entrapped in extracardiac organs after transplantation and difficult to retain in intact myocardium 31. The cardiac retention in IR group increased by 95.8% compared with that in control group, which roughly showed that the homing effect of SDF‐1α up‐regulation after myocardial ischemia. The efficacy was similar to that increase of 80.5% in infarcted hearts compared with sham‐operated group 26. In that research, SDF‐1α gene over‐expression was not associated with more stem cells mobilization to intact myocardium, as a result of increased expression of several genes after myocardial infarction, in addition to SDF‐1α. Similarly, we postulated the intact extra‐cardiac organs in the absence of ischemia stimulus could not recruit more MSCs even if higher SDF‐1α expression after RIC. So, we also did not further investigate the SDF‐1α gene expression change on lungs, spleen, liver or intact heart induced by rRIPoC.

As indicated previously, we validated that RIPoC procedure increased the MSCs retention in IR myocardium by 49.6% compared with that in IR microenvironment without any intervention measures 9. Then, MSCs retention in IR myocardium increased by 97.9% because of applying repeated RIPoC procedure with every 2 days. Furthermore, continuous RIPoC with every 1 day resulted in more MSCs retention by 159.6% in IR myocardium. There was on dose–effect relationship between MSCs retention in myocardium and the frequency of repeated ischaemic post‐conditioning when it was up to every 1 day. As it seemed that the effect was not on rise when rRIPoC was administered above every 1 day.

In one previous study during entire 4‐week period, stem cell engraftment was assessed by bioluminescence imaging showed MSCs sustained engraftment in injured myocardium without *de novo* cardiac differentiation 5. Our previous experiment also observed no differentiation or any proliferation in extra‐cardiac organs by immunohistochemistry 4. The technology of PCR could sensitively detect the SRY gene with non‐fractured segment on Y chromosome. Interestingly, a trend was seen that the distribution in heart was inversely correlated with that in extra‐cardiac organs among all the groups. The result was in accordance with the phenomenon that augmenting SDF‐1α expression in myocardium was accompanied with higher MSCs retention in the heart and lower amount in the extracardiac organs, suggested that SDF‐1α‐dominated MSCs homing to ischaemic heart existed.

Administration of anti‐CXCR4 antibody, which specifically blocks binding of SDF‐1α to its endogenous receptor CXCR4, diminished MSCs recruitment after rRIPoC with every 1 day by 71.6%, and strongly suggested a requirement of SDF‐1α by rRIPoC procedure for MSCs recruitment to the IR heart. Accordingly, cardiac function enhancement was abrogated in accordance with the phenomenon that MSCs retention increase in myocardium was blocked by SDF‐1α/CXCR4 pathway. This confirmatory experiment also demonstrated that cardiac function in group specific CXCR4‐antibody blockade with reduced MSCs retention in the condition of rRIPoC administration was inferior to that in group IR with more MSCs retention without rRIPoC administration, which indicated the MSCs was major determinant of cardiac function. The result was consistent to the study that rRIPoC administration after a single early episode of ischaemic preconditioning could not bring about further less infarct size 10.

Taken together, a potential clinical approach of superimposed effect was successfully resorted to recruit MSCs retention into myocardium in this study. Repeated RIPoC could consistently enhance SDF‐1α gene expression in ischaemic myocardium, changed MSCs distribution of the intramyocardially injected MSCs over systemic organs by SDF‐1α/CXCR4 interaction. Our study gave rise to new evidence that systemic redistribution was important for enhancing therapeutic efficacy of stem cell transplantation and pave the way for emerging investigation of recipient conditioning for MSCs transplantation and their impact on target and untargeted organs 32.

## Conflict of interest

The authors declare no conflict of interest.
